# KLF2 is a clinical diagnostic and treatment biomarker of breast cancer

**DOI:** 10.3389/fcell.2023.1182123

**Published:** 2023-04-13

**Authors:** Ya-Zhao Li, Juan Xie, Rui-Qi Wang, Xiao-Qian Gao, Pei-Jun Liu, Jie Liu

**Affiliations:** ^1^ Center for Translational Medicine, the First Affiliated Hospital of Xi’an Jiaotong University, Xi’an, Shaanxi, China; ^2^ Key Laboratory for Tumor Precision Medicine of Shaanxi Province, the First Affiliated Hospital of Xi’an Jiaotong University, Xi’an, Shaanxi, China; ^3^ Department of Clinical Laboratory, Shaanxi Provincial People’s Hospital, Xi’an, Shaanxi, China

**Keywords:** KLF2, breast cancer, immune infiltration, VEGFA-HIF1α, simvastatin

## Abstract

**Background:** As a highly prevalent malignancy among women worldwide, breast cancer, remains a critical public health issue necessitating the development of novel therapeutics and biomarkers. Kruppel Like Factor 2 (KLF2), a member of the Kruppel family of transcription factors, has been implicated in various types of cancer due to its diminished expression; however, the potential implications of KLF2 expression in relation to breast cancer progression, prognosis, and therapy remain unclear.

**Methods:** The present study employed the Tumor Immune Estimation Resource (TIMER) and The Human Protein Atlas databases to investigate the expression pattern of KLF2 in pan-cancer. The relationship between KLF2 expression and clinical features or immune infiltration of The Cancer Genome Atlas (TCGA) breast cancer samples was evaluated using Breast Cancer Integrative Platform (BCIP) and TIMER. The expression levels of KLF2 in breast cancer were validated via immunohistochemical staining analysis. Gene Set Enrichment Analysis (GSEA) to study the KLF2-related gene ontology. STRING database was employed to construct a protein-protein interaction (PPI) network of KLF2 in relation to vascular endothelial growth factor A (VEGFA) and hypoxia-inducible factor 1α (HIF1α). The expression of KLF2 following diverse breast cancer therapies was analyzed in the Gene Expression Omnibus (GEO) databases. The expression of KLF2 following treatment with simvastatin was validated via immunofluorescence and western blotting.

**Results:** Our study reveals that KLF2 displays significantly reduced expression in cancerous tissues compared to non-cancerous controls. Patients with low KLF2 expression levels exhibited poor prognosis across multiple cancer types. KLF2 expression levels were found to be reduced in advanced cancer stages and grades, while positively correlated with the expression of estrogen receptor (ER), progesterone receptor (PR), and tumor size in breast cancer. KLF2 expression is associated with diverse immune infiltration cells, and may impact the breast tumor immune microenvironment by regulating dendritic cell activation. Additionally, we observed a negative correlation between KLF2 expression levels and angiogenesis, as well as the expression of VEGFA and HIF1α. Notably, the anticancer drug simvastatin could induce KLF2 expression in both breast cancer.

**Conclusion:** Based on our observations, KLF2 has potential as a diagnostic, prognostic, and therapeutic biomarker for breast cancer.

## Introduction

Breast cancer remains a significant contributor to cancer-related deaths, accounting for 15% of all cases. Incidence rates continue to rise, with breast cancer, lung cancer, and colon and rectum making up 52% of all new diagnoses, and breast cancer alone representing 31% of female cancers. (Giaquinto et al., 2022; Siegel et al., 2023). As such, identifying sensitive diagnostic markers and therapeutic targets is crucial for mitigating the health risks of breast cancer.

Kruppel-like factor 2 (KLF2), a transcription factor family member with conserved zinc-finger domains, (Rane et al., 2019), has been extensively studied in various biological processes, including lung development, erythropoiesis, hemodynamic regulation, lymphocyte development, and T-cell survival (Kuo et al., 1997; Feinberg et al., 2004; Wang et al., 2022). Recent studies have revealed its involvement in the genesis and progression of several cancers, including clear cell renal cell carcinoma (Lu et al., 2021), gastric Cancer (Wang et al., 2022), colorectal cancer (Zeng et al., 2018; Osman et al., 2021), lung cancer (Ma et al., 2021), hepatocellular carcinoma (Qu et al., 2022), *etc.* Furthermore, recent research has demonstrated that KLF2 functions as a tumor suppressor in breast cancer (Zhu et al., 2022). However, whether KLF2 can serve as a potential biomarker for prognostic prediction and therapy remains unclear.

In this study, we analyzed The Cancer Genome Atlas (TCGA) breast cancer and Gene Expression Omnibus (GEO) breast cancer cohort using online resources. We employed bioinformatics tools and online websites to investigate the differential expression of KLF2 in pan-cancer. We validated the differential expression of KLF2 in breast cancer and normal tissue samples using immunohistochemical (IHC) staining. A comprehensive analysis of KLF2 co-expression gene networks in breast cancer was conducted to explore the underlying biological functions and signaling pathways associated with these genes. Furthermore, in breast cancer, we explored the association between KLF2 and diverse immune infiltration cells, angiogenesis, vasculature development, and endothelium development, and DNA double-strand break repair, which may be related to the VEGF-Hif1α pathway. Our analysis showed that KLF2 expression increased after statin treatment and neoadjuvant trial based on the GEO database and our experimental data. These findings provide a basis for KLF2 to potentially serve as a new diagnostic and therapeutic target in breast cancer.

## Materials and methods

### Expression of KLF2 in caners and tumor immune infiltrating cells

The study utilized the Tumor Immune Estimation Resource (TIMER) database, which encompasses 10,897 samples from 32 cancer types in The Cancer Genome Atlas (TCGA) (Li et al., 2017), to investigate the expression of KLF2 across different cancer types and its correlation with immune infiltrates such as T cells, B cells, dendritic cells, macrophages, and neutrophils. Gene expression levels were evaluated with respect to tumor purity and displayed using box plots, with statistical significance assessed using the Wilcoxon test. Additionally, the somatic copy number alteration module of the TIMER tool was utilized to investigate the correlation between genetic copy number variation (CNV) of KLF2 and the proportion of immune infiltrating cells in breast cancer. The study also employed the “Correlation” module to examine the expression correlation between KLF2 and immune infiltration marker genes. This comprehensive analysis provides important insights into the role of KLF2 in cancer immune infiltration and may offer novel therapeutic strategies for cancer treatment (Liu et al., 2022).

The Human Protein Atlas (The Human Protein Atlas, https://www.proteinatlas.org) project, which was initiated in 2003 in Sweden, aims to map all human proteins across various cells, tissues, and organs through an integrated approach involving a multitude of omics techniques, such as mass spectroscopy-based proteomics, transcriptomics, antibody-based imaging, and systems biology (Uhlen et al., 2017). This atlas provides a resource for immunostaining of tissues and cell lines, as well as analysis of differential protein expression in both normal and tumor tissues. KLF2 immunohistochemical results for normal breast, kidney, endometrium, stomach, colon, lung, and thyroid gland tissues, along with tumor tissues, are available on the website.

### KLF2 gene Co-Expression with pathologic characteristic of breast cancer

The Breast Cancer Integrative Platform (BCIP, http://www.omicsnet.org/bcancer/) is an integrative platform that employs uniform normalization methods to ensure strict quality control of multi-omics data. The BCIP database comprises 9005 breast tumors and 376 adjacent non-cancerous breast tissues sourced from the NCBI Gene Expression Omnibus (GEO) (Song et al., 2021). Herein, we utilize this platform to perform an integrative analysis of the relationship between KLF2 gene expression and patient survival, pathologic stage, ER/PR/HER2 status, tumor size and prognosis in different breast cancer subtypes.

### Immunohistochemistry

Ethical approval for the use of 33 pairs of paracancer and breast cancer tissues was granted by the Human Research Ethics Committee of the First Affiliated Hospital of Xi’an Jiaotong University in Shaanxi, China. Written informed consent was obtained from all patients. Female BALB/c mice (4–6 weeks old, n = 6) and C57BL/6J mice (4–6 weeks old, n = 6) were procured from Xi’an Jiaotong University and all animal procedures were conducted according to the approved protocol by the Institutional Animal Care and Use Committee at Xi’an Jiaotong University.

Immunohistochemistry and quantification procedures were carried out following previously described methods (Wang et al., 2019; Liu et al., 2020). Briefly, tissues were fixed using 10% formaldehyde and paraffin-embedded. Sections were subsequently prepared and incubated with primary antibodies against KLF2 (Abcam, United Kingdom, 1:500). Images of the stained sections were captured using a Leica SCN400 slide scanner (Leica, Germany). The immunohistochemical staining results were individually evaluated in a blinded manner by three pathologists with no access to the clinical or pathological status of the specimens. The staining intensity was graded on a scale of 0 (negative), 1 (weakly positive), 2 (moderately positive), or 3 (strongly positive). The staining intensity was assessed on a scale of 0 (negative), 1 (1%–25%), 2 (26%–50%), 3 (51%–75%), or 4 (76%–100%), based on the proportion of positive cells. The staining score for each field was calculated by multiplying the intensity and extent of the staining. The final staining score for each sample was determined as the mean score of three randomly selected fields. Negative expression level was assigned to samples with final scores ranging from 0–2, while positive expression level was assigned to samples with final scores ranging from 3–12. Samples with final scores ranging from 3-5 were classified as low expression, while those with final scores ranging from 6–12 were classified as high expression.

### Gene Set Enrichment Analysis

To investigate the potential role of KLF2 in breast cancer, we stratified samples from the breast cancer cohort into high and low expression groups based on the median KLF2 expression level in breast cancer samples. We then performed Gene Set Enrichment Analysis (GSEA) (www.gsea-msigdb.org/gsea/index.jsp) (Subramanian et al., 2005) to identify whether genes in these two groups were enriched for specific biological functions.

### Protein-protein interaction (PPI) network of KLF2

The STRING database (https://string-db.org/) is a comprehensive resource that integrates known and predicted associations between proteins, including both physical interactions and functional associations. This is achieved through evidence collection and scoring from multiple sources, including automated text mining of scientific literature, interaction experiments and annotated complexes/pathways databases, computational interaction predictions from co-expression and from conserved genomic context, and systematic transfers of interaction evidence across different organisms. The database strives for wide coverage and the upcoming version 11.5 will contain more than 14,000 organisms (Szklarczyk et al., 2021). In this study, we conducted an analysis of the interaction between KLF2, HIF1α, and VEGFA using the STRING database.

### KLF2 expression after different therapy of breast cancer

The Gene Expression Omnibus (GEO), available at www.ncbi.nlm.nih.gov/geo, is an open-access public repository that hosts functional genomics data that adhere to the Minimum Information About a Microarray Experiment (MIAME) guidelines. This repository accepts both array- and sequence-based data. Tools are provided to aid users in querying and retrieving experiments as well as curated gene expression profiles (Barrett et al., 2013). In this study, we conducted an analysis of KLF2 expression following treatment with atorvastatin (GSE63427) or in the context of a neoadjuvant trial (GSE114403).

### Western blotting

Cells that had undergone treatment were disrupted using RIPA buffer, after which the cell lysates were resolved by SDS-PAGE and subsequently transferred onto a PVDF membrane. The PVDF membrane was then blocked with 5% fat-free milk in TBST prior to being probed with primary antibody, followed by incubation with a HRP-conjugated secondary antibody (Proteintech, China, 1:10000). Detection of the chemiluminescent signals was carried out utilizing the ChemiDocTM XRS+ (Bio-rad, United States). The primary antibodies that were used in this study include anti-KLF2 (1:1000), and β-actin (Proteintech, China, 1:10000).

### Immunofluorescence

Mouse tissues were fixed with 4% paraformaldehyde (PFA) and permeabilized in phosphate-buffered saline (PBS) containing 0.5% Triton X-100. Blocking was performed using 5% bovine serum albumin (BSA) supplemented with 10% goat serum. Subsequently, samples were incubated with primary antibodies against KLF2 (Abcam, United Kingdom, 1:500). Nuclei were counterstained with 4, 6-diamidino-2-phenylindole (DAPI; 5 μg/mL). Confocal images were acquired using a Leica TCS SP5 confocal laser scanning microscope (Leica, Germany).

### Statistical analysis

Statistical analyses were carried out using Prism 7.0 software. Results are presented as the mean ± standard error of the mean (SEM). Significance was determined by performing a two-tailed Student’s t-test or two-way ANOVA test. All experiments were performed in triplicate, and a *p*-value of less than 0.05 was considered statistically significant.

## Results

### Pan-cancer analysis of KLF2 expression level

**FIGURE 1:**
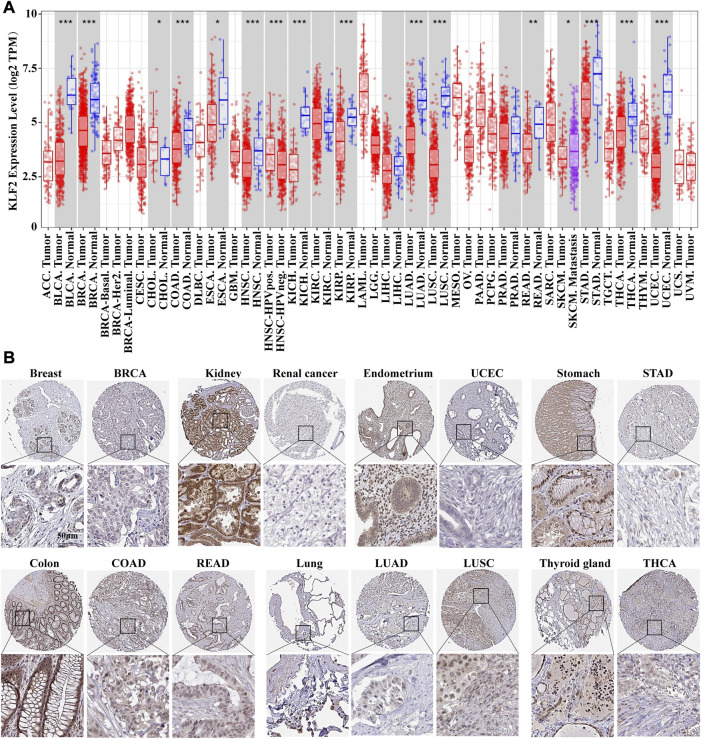
The expression of KLF2 in breast cancer and pan-cancer**. (A)** The expression of KLF2 in pan-cancer was evaluated using the TIMER database. **(B)** To investigate the protein expression of KLF2, an immunohistochemistry assay was performed on both paracarcinoma and tumor samples.

The mRNA expression differences of KLF2 in various cancers were analyzed using the TIMER tool. The results of the database analysis showed that KLF2 expression was predominantly lower in tumor tissues than in their corresponding normal tissues in several cancer types, such as bladder urothelial carcinoma (BLCA), breast invasive carcinoma (BRCA), cervical and endocervical (CESC), colon adenocarcinoma (COAD), esophageal carcinoma (ESCA), head and neck squamous cell carcinoma (HNSC), kidney chromophobe (KICH), kidney renal papillary (KIRP), lung adenocarcinoma (LUAD), lung squamous cell carcinoma (LUSC), prostate adenocarcinoma (PRAD), rectum adenocarcinoma (READ), stomach adenocarcinoma (STAD), thyroid carcinoma (THCA), and uterine corpus endometrial carcinoma (UCEC). Notably, in cholangio carcinoma (CHOL), KLF2 expression was higher in tumor tissue than in normal tissue (Figure 1A).

The Human Protein Atlas was utilized to evaluate KLF2 expression in various cancers. KLF2 was predominantly expressed at high levels in normal tissue than their tumor tissues, such as, breast, kidney, endometrium, stomach, colon, lung, and thyroid gland tissues in TCGA database (Figure 1B). Moreover, we examined the effect of KLF2 dysregulation on patient survival rates and found that only patients with adrenocortical carcinoma (ACC), kidney renal clear cell carcinoma (KIRC), and sarcoma (SARC) had increased survival rates with high KLF2 expression. In other tumor types, patients with high KLF2 expression exhibited a trend of extended survival, albeit without statistical significance (Supplementary Figure S1). Hence, our data suggest that KLF2 may serve as a tumor suppressor gene in the majority of common tumors.

### Expression levels of KLF2 in patients with breast cancer

**FIGURE 2:**
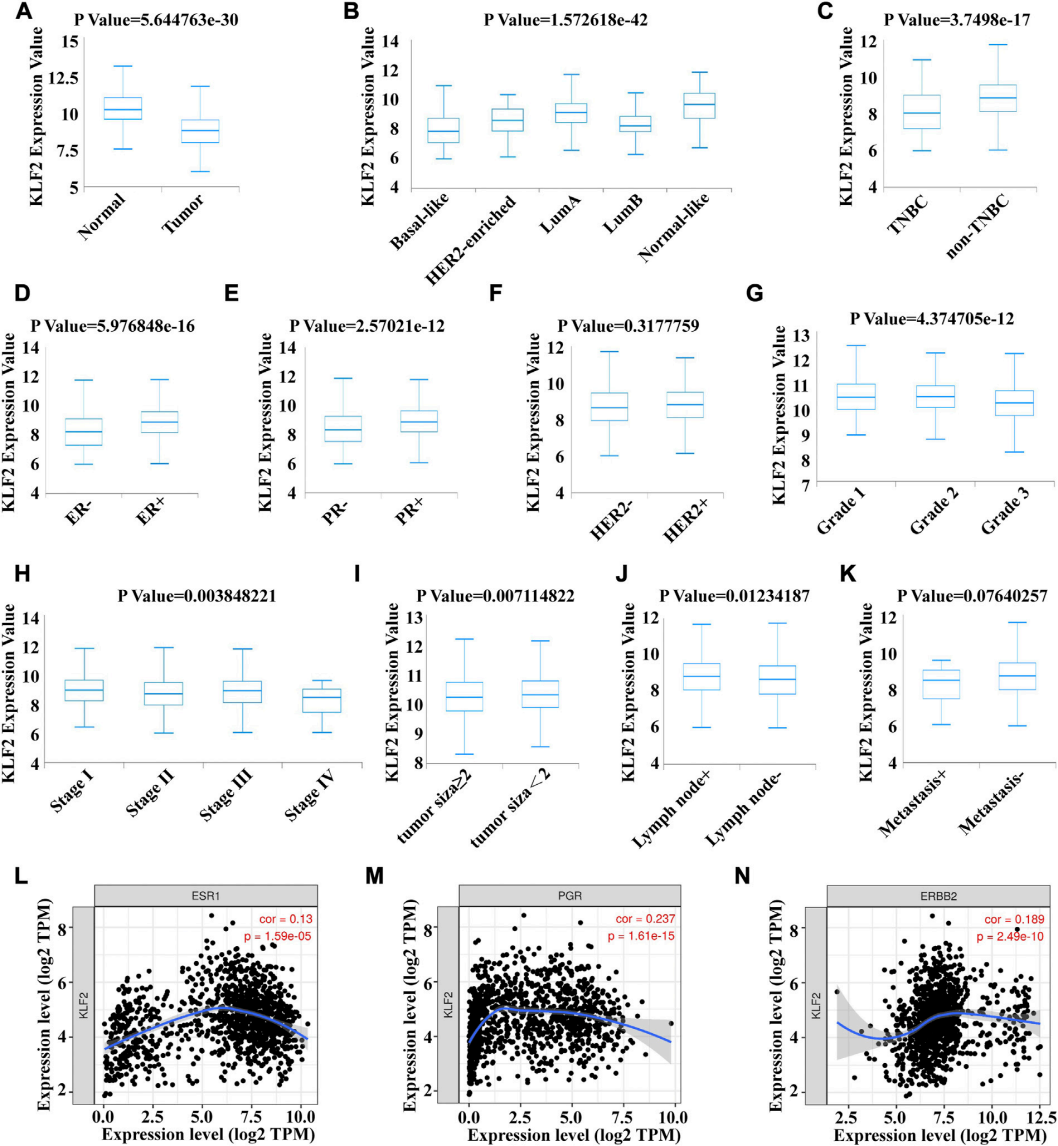
Correlation between KLF2 mRNA expression and various clinicopathological parameters in patients with breast cancer. The KLF2 mRNA expression level were determined using BCIP in **(A)** normal and tumor tissue, **(B)** subtypes, **(C)** TNBC and non-TNBC, **(D)** ER status, **(E)** PR status, **(F)** HER2 status, **(G)** pathologic grade, **(H)** pathologic stage, **(I)** tumor size, **(J)** lymph node metastases and **(K)** distant metastasis. Association between the expression level of KLf2 and ER **(L)**, PR **(M)** and HER2 **(N)**. *, *p* < 0.05; **, *p* < 0.01; ***, *p* < 0.001; ns, no significant.

**FIGURE 3:**
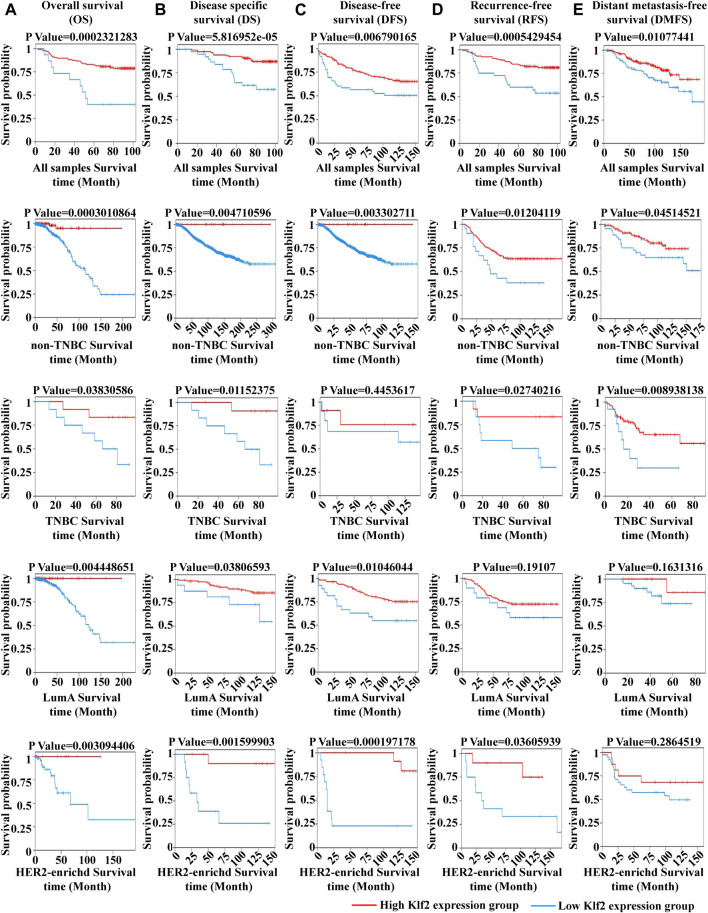
The impact of KLF2 on the survival probability of breast cancer patients. Overall survival (OS) **(A)**, disease specific survival (DS) **(B)**, disease-free survival (DFS) **(C)**, recurrence-free survival (RFS) **(D)**, and distant metastasis-free survival (DMFS) **(E)**.

**FIGURE 4:**
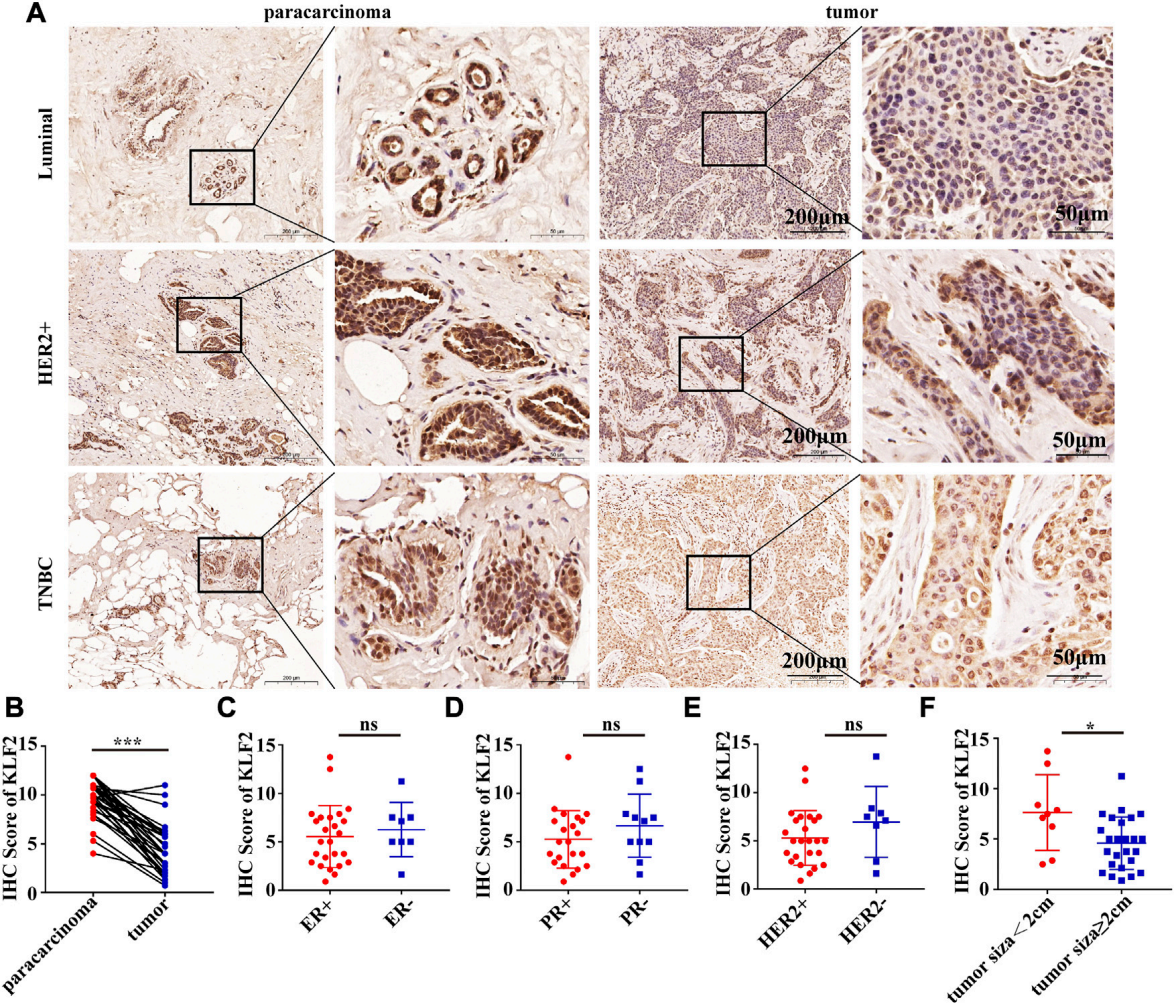
KLF2 protein was lower expressed in breast cancer than paracarcinoma tissues. **(A, B)** Immunohistochemistry was performed to evaluate the expression of KLF2 in paracancerous and breast cancer tissues. Relationship between the level of KLF2 expression and ER **(C)**, PR **(D)**, HER2 **(E)** and tumor size **(F)**. *, *p* < 0.05; **, *p* < 0.01; ***, *p* < 0.001; ns, no significant.

To verify the clinical significance of KLF2, we analyzed the clinical data from the TCGA breast cancer cohort. Through analysis of breast cancer datasets from TCGA in BCIP, we determined the difference in KLF2 expression between breast cancer and normal samples. Our analysis demonstrated that the expression level of KLF2 was significantly reduced in breast cancer compared to the control group (Figure 2A). Additionally, KLF2 expression decreased from normal-like, luminal, HER2-enriched to basal-like in the different subtypes of breast cancer tissues (Figure 2B), and was highly expressed in non-triple negative breast cancer (TNBC) than in TNBC (Figure 2C). Furthermore, KLF2 showed a positive correlation with ER and PR expression status (Figures 2D, E), and a positive correlation with HER2 status, although it was not statistically significant (Figure 2F). We observed a downward trend in KLF2 expression with the increase of pathological grade and pathological stage (Figures 2G, H), and KLF2 expression exhibited a negative correlation with tumor size, lymph node metastases, and distant metastasis (Figures 2I–K). Additionally, we found that KLF2 expression was positively correlated with ER (r = 0.13, *p* = 1.59e-05), PR (r = 0.237, *p* = 1.61e-15), and HER2 (r = 0.189, *p* = 2.49e-10) expression in TCGA by analyzing with TIMER (Figures 2L–N). We further explored whether KLF2 could serve as a prognostic indicator and found that patients with high KLF2 expression in all subtypes had better overall survival (OS) (Figure 3A), disease-specific survival (DS) (Figure 3B), disease-free survival (DFS) (Figure 3C), recurrence-free survival (RFS) (Figure 3D), and distant metastasis-free survival (DMFS) (Figure 3E) than patients with low expression. To validate these results in the database, the IHC staining results indicated that KLF2 was remarkably lower than that in paracancerous tissues (Figures 4A, B; Table 1). Although there was no significant correlation between KLF2 and the expression of ER, PR, and HER2 in the analysis, which may be caused by the small sample size (Figures 4D–F; Table 1), a significant negative correlation was found between KLF2 and tumor size (Figure 4G; Table 1). Taken together, KLF2 may serve as a marker for the clinical diagnosis of breast cancer.

**TABLE 1:** Associations between KLF2 expression levels and clinic-pathologic parameters in breast cancer patients.

Variables	No.	KLF2 expression levels		*p*-Value
		Low	High	
KLF2				
Paracarcinoma	33	2	31	<0.0001
Tumor	33	22	11	
ER				
+	25	19	6	0.5760
-	8	5	3	
PR				
+	22	17	5	0.2220
-	11	6	5	
HER2				
+	25	19	6	0.1879
-	8	4	4	
Tumor size (cm)				
<2	9	2	7	0.0156
≥2	24	20	4	

### Associations between KLF2 and tumor immune infiltrating cells

**FIGURE 5:**
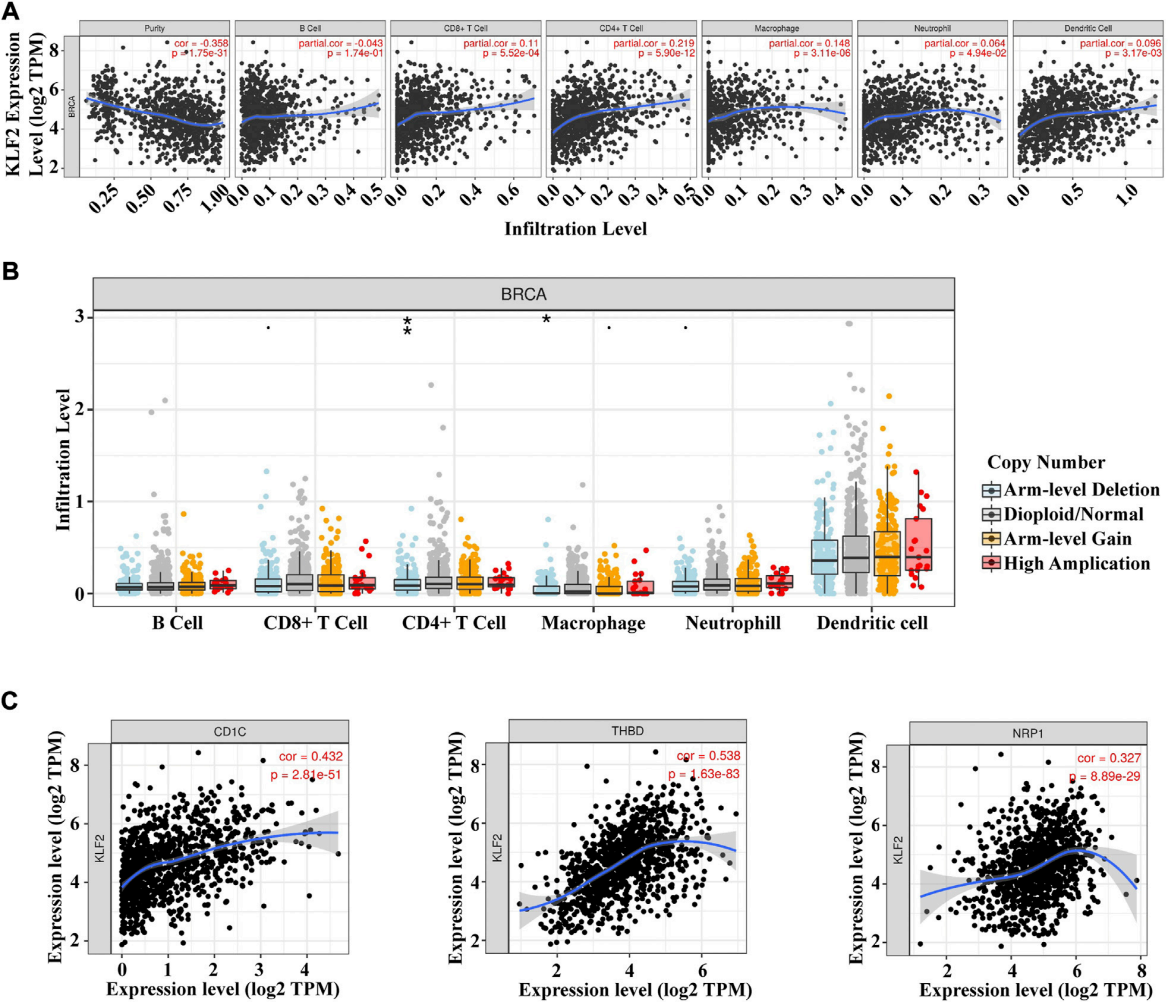
Relationships between KLF2 and tumor immune infiltrating cells. **(A)** Association between the expression level of KLF2 and immune infiltration cells in breast cancer. **(B)** KLF2 CNV affects the infiltrating levels of B cell, CD8^+^ T cell, CD4^+^ T cell macrophages, neutrophils, and dendritic cell in breast cancer. **(C)** The correlation between KLF2 expression and dendritic cells in breast cancer. ^∗^, *p* < 0.05; ^∗∗^, *p* < 0.01; ^∗∗∗^, *p* < 0.001; ns, no significant.

The present study analyzed the correlation between KLF2 expression levels and immune cell infiltration in breast cancer samples using the TIMER database. The findings revealed that KLF2 expression level had a positive association with the infiltrating levels of CD8^+^ T cells (r = 0.11, *p* = 5.52E-4), CD4^+^ T cells (r = 0.219, *p* = 5.90E-12), macrophages (r = 0.148, *p* = 3.17E-2), neutrophils (r = 0.064, *p* = 4.94E-2), and dendritic cells (DCs) (r = 0.098, *p* = 3.17E-3) in breast cancer samples (Figure 5A). Furthermore, the study observed a significant correlation between KLF2 CNV and infiltration levels of CD4^+^ T cells and macrophages in breast cancer patients (Figure 5B). These findings suggest that KLF2 may play a role in regulating the immune microenvironment in breast cancer.

To further assess the relationship between KLF2 and immune cell infiltration in breast cancer, we conducted an analysis of the TIMER database to explore the correlation between KLF2 and immune cell marker genes in different types of immune cells. Our results demonstrated that KLF2 expression was positively correlated with the majority of immune cell marker genes, particularly those associated with dendritic cells, such as CD1C (r = 0.432, *p* = 2.81e-51), THBD (r = 0.538, *p* = 1.63e-83), and NRP1 (r = 0.327, *p* = 8.89e-29). Correspondingly, the scatter plots depicted a significant positive correlation between KLF2 expression level and dendritic cell immune marker genes, respectively (Figure 5C; Table 2). These findings suggest that KLF2 may play a role in regulating the dendritic cell activation in breast cancer.

**TABLE 2:** Correlation analysis between KLF2 and immune cell marker gene in TIMER database.

Description	Gene markers	rho	*p*-Value
B cell	CD19	0.225	**4.5e-14**
	MS4A1	0.297	**6.9e-24**
CD8 + T Cell	CD8A	0.26	**1.7e-18**
	CD8B	0.204	**9.28e-12**
	IL2RA	0.01	0.752
Tfh	CXCR3	0.277	**7.52–21**
	CXCR5	0.251	**2.64e-17**
	ICOS	0.076	**1.17e-02**
Th1	IL12RB1	0.198	**3.49e-11**
	CCR1	0.005	0.861
	CCR5	0.172	**9.97e-09**
Th2	CCR4	0.254	**1.08e-17**
	CCR8	0.004	0.892
	HAVCR1	0.042	0.167
Th17	IL21R	0.153	**3.59e-07**
	IL23R	0.104	**5.21e-04**
	CCR6	0.313	**2.22e-26**
Treg	FOXP3	0.069	**2.27e-02**
	NT5E	0.192	**1.28e-10**
	IL7R	0.254	**1.3e-17**
T cell exhaustion	PDCD1	0.213	**1.06e-12**
	CTLA4	0.079	**8.42e-03**
	LAG3	−0.022	0.461
M1 Macrophage	NOS2	0.175	**5.35e-09**
	IRF5	0.119	**7.72e-05**
	PTGS2	0.29	**9.78e-23**
M2 Macrophage	CD163	0.037	0.219
	MRC1	0.199	**3.05e-11**
	CD209	0.193	**1.04e-10**
TAM	CCL2	0.182	**1.09e-09**
	CD86	0.111	**2.16e-04**
	CD68	0.109	**2.96e-04**
Monocyte	CD14	0.146	**1.11e-06**
	CD33	0.258	**4.96e-22**
	ITGAX	0.235	**3.23e-15**
Natural killer cell	B3GAT1	0.081	**7.06e-03**
	KIR3DL1	0.079	**8.71e-03**
	CD7	0.203	**1.08e-11**
Neutrophil	FCGR3A	0.02	0.508
	CD55	0.071	**1.85e-02**
	ITGAM	0.193	**1.07e-10**
Dendritic cell	CD1C	0.432	**2.81e-51**
	THBD	0.538	**1.63e-83**
	NRP1	0.327	**8.89e-29**

Significant *p* values (*p*< 0.05) are indicated in bold.

The bold values indicate significantly changed markers.

### Gene Set Enrichment Analysis

**FIGURE 6:**
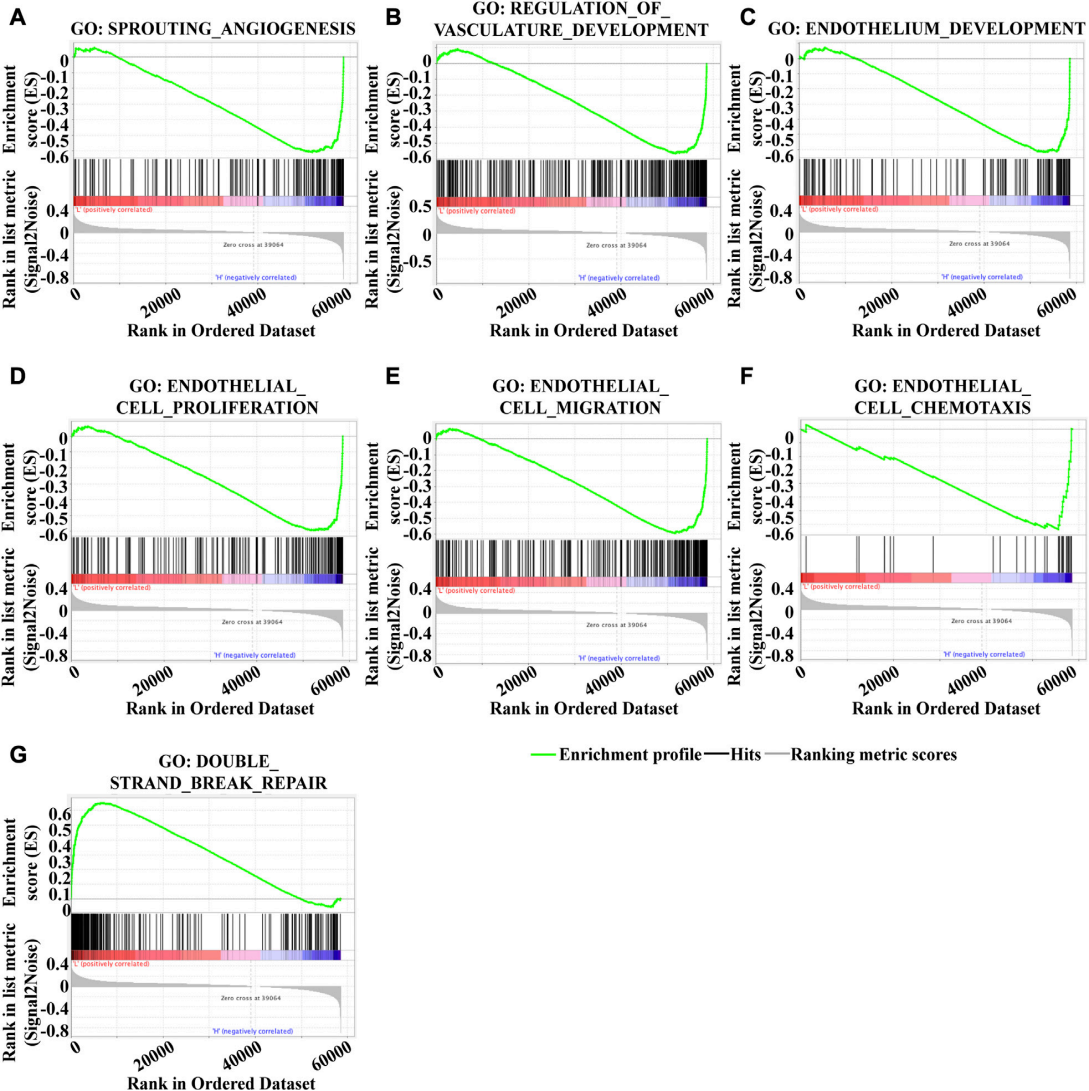
Gene set enrichment analysis of GO analysis. Sprouting _angiogenesis **(A)**, Regulation_of_vasculature_development **(B)**, Endothelium_development **(C)**, Endothelial_cell_proliferation **(D)**, Endothelial_cell_migration **(E)**, Endothelial_cell_chemotaxis **(F)**, and Double_strand_break_repair **(G)**.

**FIGURE 7:**
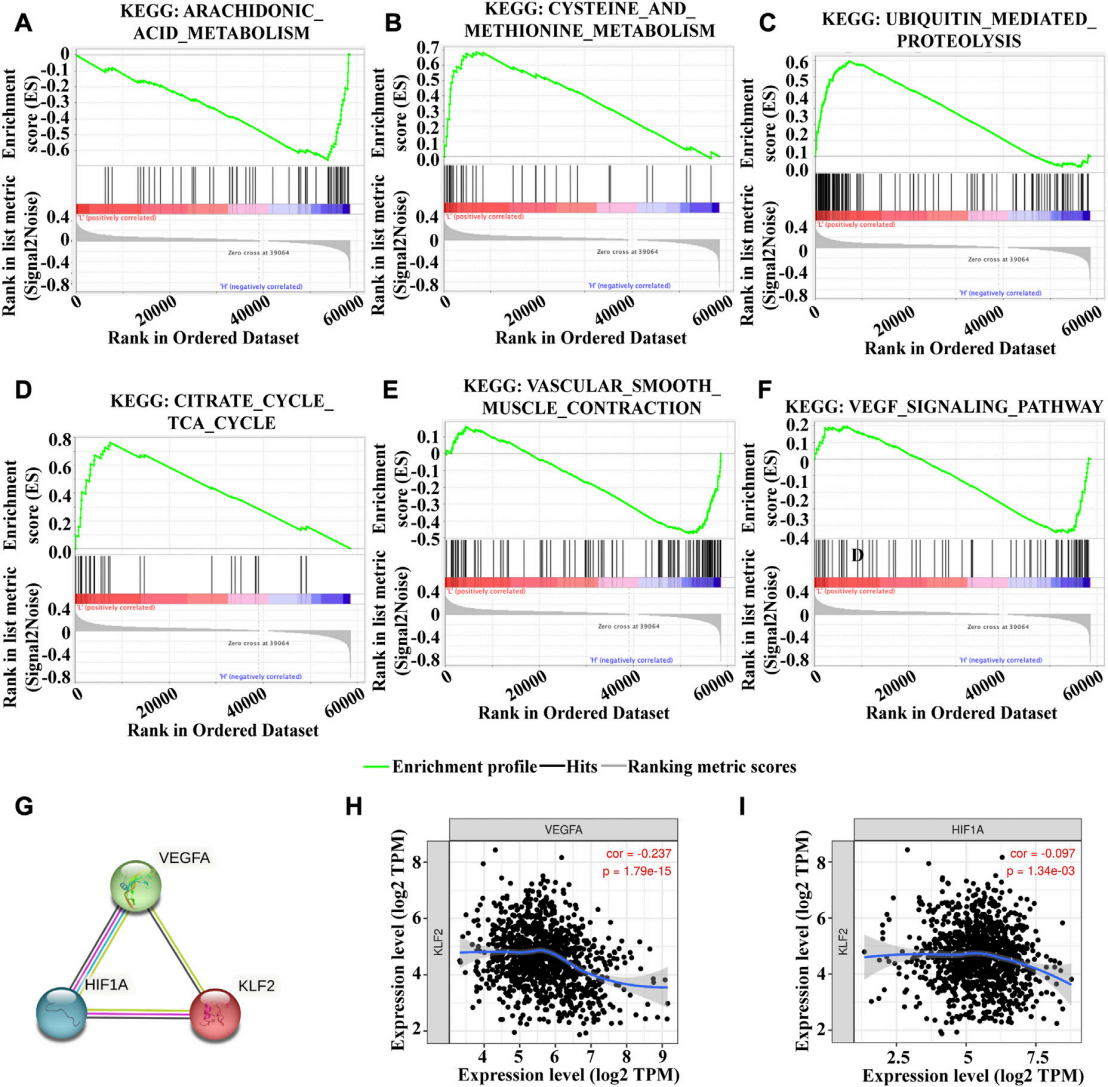
Gene set enrichment analysis of KEGG analysis. Arachidonic_acid_metabolism **(A)**, Cysteine_and_methionine_metabolism **(B)**, Ubiquitin_mediated_proteolysis **(C)**, Citrate_cycle_tca_cycle **(D)**, Vascular_smooth_muscle_contraction **(E)** and VEGF_signaling_pathway **(F)**. **(G)**. PPI analysis of KLF2, VEGFA and HIf1α. KLF2 expression correlated with VEGFA **(H)** and HIf1α **(I)** in breast cancer.

To investigate the underlying mechanism of KLF2 in breast cancer, we performed Gene Set Enrichment Analysis (GSEA) to identify differentially expressed genes. Gene Ontology (GO) analysis revealed significant changes in biological processes related to angiogenesis, such as sprouting_angiogenesis, regulation_of_vasculature_development, endothelium_development, endothelial_cell_proliferation, endothelial_cell_migration, and endothelial_cell_chemotaxis (Figures 6A–F), as well as in double_strand_break_repair (Figure 6G). Moreover, KLF2 expression was associated with several metabolic pathways in the KEGG enrichment analysis, including arachidonic_acid_metabolism, cysteine_and_methionine_metabolism, ubiquitin_mediated_proteolysis, and citrate_cycle_tca_cycle (Figures 7A–D). Conversely, KLF2 expression was negatively correlated with pathways related to angiogenesis, including vascular_smooth_muscle_contraction and VEGF_signaling_pathway (Figures 7E, F). Analysis of the STRING database suggested that KLF2 may interact with VEGFA and HIF1α (Figure 7G). The scatter plots showed a negative correlation between KLF2 expression level and VEGFA (r = −0.237, *p* = 1.79e-15) and HIF1α (r = −0.097, *p* = 1.34e-03) (Figures 7H, I).

### Associations of KLF2 expression level with clinical treatment in breast cancer

**FIGURE 8:**
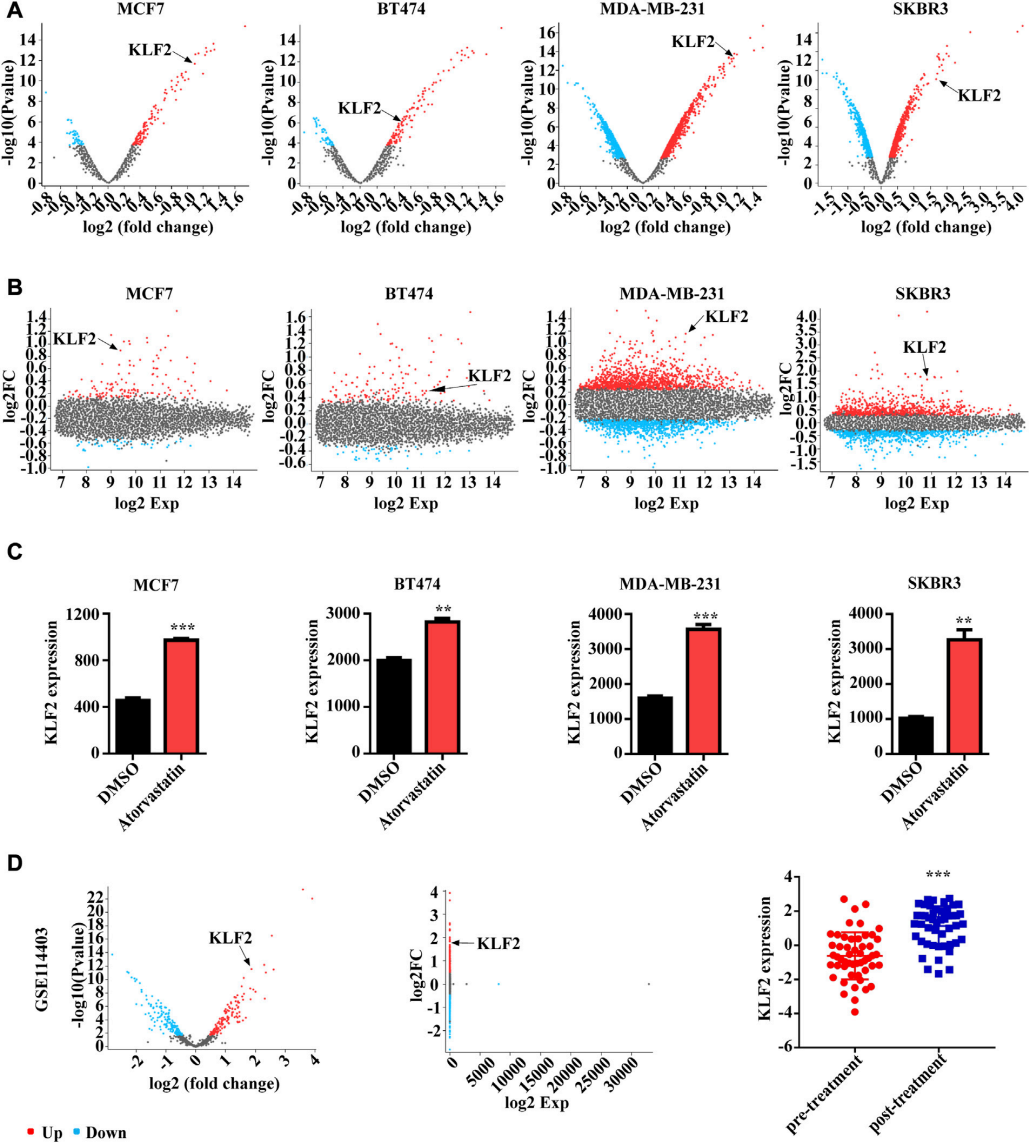
The relationship between KLF2 expression levels and clinical treatment in breast cancer. The volcano plot **(A)** and meandiff plot **(B)** depict the atorvastatin-induced transcriptional changes in breast cancer cells, MCF7, BT474, MDA-MB-231 and SKBR3. **(C)** KLF2 expression value after atorvastatin treatment in GSE63427. **(D)** The volcano plot, meandiff plot and KLF2 expression in GSE114403. ^∗^, *p* < 0.05; ^∗∗^, *p* < 0.01; ^∗∗∗^, *p* < 0.001; ns, no significant.

In order to investigate the potential role of KLF2 in the clinical management of breast cancer, we conducted an analysis of the GSE63427 dataset obtained from the GEO database. This dataset reports transcriptional changes induced by statin treatment in breast cancer, and we found that KLF2 expression was upregulated in MCF7 (Luminal A subtype), BT474 (Luminal B subtype), SKBR3 (HER2 subtype) and MDA-MB-231 (Basal subtype) cells after atorvastatin treatment compared to control cells. Specifically, KLF2 expression was among the top 10 upregulated genes in MCF7 and MDA-MB-231 cells following treatment with atorvastatin (Figures 8A, B). Furthermore, KLF2 expression was increased more than 2-fold in MCF7 and MDA-MB-231 cells and more than 3-fold in SKBR3 cells, compared to their respective control cells (Figure 8C). We also analyzed the GSE114403 dataset, which includes immune profiling of pre- and post-treatment breast cancer tissues from the SOWG S0800 randomized neoadjuvant trial of weekly nab-paclitaxel with or without bevacizumab and dose dense doxorubicin and cyclophosphamide. Our analysis showed that KLF2 expression was upregulated in post-treatment samples compared to pre-treatment samples (Figure 8D). These findings suggest that increased expression of KLF2 may serve as a potential marker of therapeutic effect.

### Simvastatin induces KLF2 expression *in vivo* and *in vitro*

**FIGURE 9:**
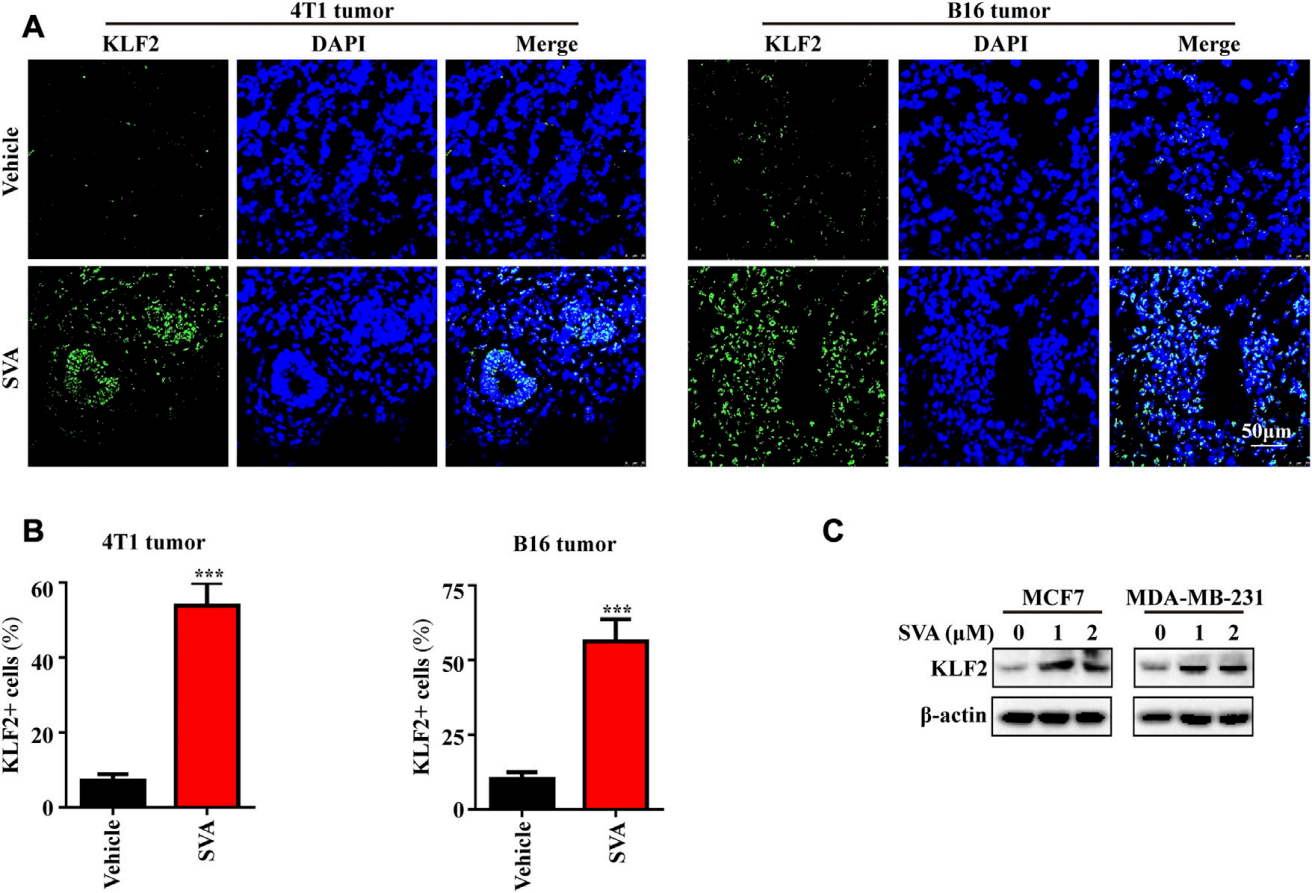
The induction of KLF2 expression by Simvastatin. **(A, B)** Immunofluorescence analysis of KLF2 was conducted on 4T1 tumors and B16 tumors from the vehicle and 15 mg/kg/day SVA groups. Bar = 50 μm. **(C)** To examine KLF2 expression in MCF7 and MDA-MB-231 cells after treatment with 0, 1 and 2 μM SVA by western blotting. ^∗^, *p* < 0.05; ^∗∗^, *p* < 0.01; ^∗∗∗^, *p* < 0.001; ns, no significant.

Based on our previous investigations, simvastatin has been shown to effectively inhibit the proliferation of tumor cells, including breast cancer and liver cancer cells, as well as impeding the growth of patient-derived organoids (PDOs), mammary and melanoma tumors in mice (Li et al., 2017; Wang et al., 2018; Liu et al., 2023). Consequently, we analyzed the expression of KLF2 in mouse tumor tissue treated with simvastatin, which exhibited a significant increase compared to the control group (Figures 9A, B). Additionally, our findings indicate that simvastatin can induce KLF2 expression in MCF7 and MBA-MB-231 cells (Figure 9C), highlighting KLF2 as a promising target of simvastatin treatment.

## Discussion

Kruppel-like factors (KLFs) belong to the zinc finger family of DNA-binding transcription factors, and participate in a range of biological processes, such as quiescence, proliferation, differentiation, development, growth, and inflammation (Feinberg et al., 2004; McConnell et al., 2010).

Recent evidence suggests that KLF2 plays a crucial role in regulating immune cell function and various physiological and pathological conditions. KLF2 is implicated in regulating adipogenesis, inflammatory diseases such as rheumatoid arthritis and vascular diseases, chronic infections, and malignancies, including ccRCC, endometrial cancer, CRC, non-small cell lung cancer, and pancreatic cancer (Novodvorsky et al., 2014; Zeng et al., 2018; Osman et al., 2021; Wang et al., 2022). Particularly, KLF2 exerts a regulatory role in tumor progression, such as inhibiting migration and invasion in ccRCC and endometrial cancer (Lu et al., 2021; Wang et al., 2021), regulating angiogenesis in CRC and non-small cell lung cancer (Zeng et al., 2018; Ma et al., 2021), inducing senescence in pancreatic cancer (Yuedi et al., 2020), regulation of maturity of and regulating T _reg_ cells’ maturity in colon cancer (Osman et al., 2021). These findings suggest that KLF2 could serve as a potential candidate for targeted therapy and cancer diagnosis. In the present study, we have identified that KLF2 is highly expressed in 15 types of tumors compared to normal tissue, which is in agreement with previous findings (Zhu et al., 2022), and may function as a tumor suppressor gene in the majority of common tumors. Specifically, KLF2 was found to be downregulated in breast cancer and was positively correlated with the expression of ER, PR, HER2, and negatively correlated with pathological grade, pathological stage, tumor size, lymph node metastases, and distant metastasis. We observed similar results in 33 pairs of paracancer and breast cancer tissues, but further investigation with a larger sample size is needed to confirm the relationship between KLF2 and ER, PR, and HER2 in clinical tissue samples. Therefore, our findings suggest that KLF2 may function as a tumor suppressor in breast cancer, consistent with previous reports (Hu et al., 2019; Yu et al., 2022).

Previous reports have highlighted the important role of KLF2 in regulating the tumor immune microenvironment (Rabacal et al., 2016; Tang et al., 2017; Osman et al., 2021; Qu et al., 2022), while immunotherapy represents a significant hope for breast cancer treatment (Emens, 2018; Nederlof et al., 2023). Therefore, we analyzed the relationship between KLF2 and tumor immune infiltration in the TIMER database, and found that it was highly correlated with the expression of most immune marker proteins, and the most significant is correlated with dendritic cell immune marker genes, including CD1C, THBD and NRP1. Recently published research suggests that KLF2 deficiency enhances surface expression of costimulatory molecules CD40 and CD86 in DCs, promoting increased heightened vascular inflammation evidenced by increased DC presence within lesions, enhanced T cell activation and cytokine production (Alberts-Grill et al., 2016). Based on our findings, we speculate that KLF2 regulates the activation of dendritic cells, thereby enhancing the function of human T lymphocytes and playing an anti-tumor role in breast cancer. However, further studies are required to confirm this hypothesis, including investigations into the relationship between KLF2 and dendritic cells, and to provide a basis for their use as an immunotherapeutic target.

KLF2 is a molecular pattern associated with vascular homeostasis that regulates the expression of a wide range of anti-inflammatory, antioxidant, and antithrombotic genes in endothelial cells (Niu et al., 2019; Yang et al., 2022). KLF2 is also involved in regulating tumor angiogenesis (Bhattacharya et al., 2005; Renz et al., 2015; Ma et al., 2021), and in this study, we found that KLF2 is closely related to angiogenesis and endothelial function in breast cancer. Our analysis of KEGG pathways revealed a negative correlation and protein-protein interaction between KLF2, VEGFA, and HIF1α. VEGFA is a primary factor driving expansion of the tumor vascular bed and is produced by hypoxic tumor cells (Ferrara, 2004; Claesson-Welsh et al., 2013). Our findings suggest that KLF2 may inhibit tumor angiogenesis and growth in breast cancer by inhibiting the VEGFA-HIF pathway, and thus KLF2 may be an important anti-angiogenesis therapeutic target for breast cancer.

Notably, our study also found that KLF2 expression was significantly increased after atorvastatin and neoadjuvant therapy. Consistent with previous studies (Parmar et al., 2005), we also found that statins promote KLF2 expression in mouse tumor tissues and human breast cancer cells MCF7 and MDA-MB-231. Non-etheless, one of the limitations of this study is the scarcity of experimental data to comprehensively validate the specific regulatory mechanism of KLF2 in anti-tumor therapy. In addition, we discovered that KLF2 can regulate multiple metabolic pathways, including arachidonic acid metabolism, cysteine and methionine metabolism, ubiquitin-mediated proteolysis, and TCA cycle. Simvastatin, a first-line drug used to treat hyperlipidemia, has been found to regulate metabolism in multiple tumors (Brown et al., 2014; Jin et al., 2019; Feng et al., 2020). In addition, we found that KLF2 can regulate DNA double-strand break repair. Our previous study showed that simvastatin can induce DNA double-strand break repair (Li et al., 2017), and we speculate that simvastatin may affect DNA double-strand break repair in tumor cells by up-regulating KLF2 expression. In addition, we found that KLF2 can regulate DNA double-strand break repair. Our previous study showed that simvastatin can induce DNA double-strand break repair, and we speculate that simvastatin may affect DNA double-strand break repair in tumor cells by up-regulating KLF2 expression (Li et al., 2017). Our RNA-seq data of simvastatin-treated breast cancer cells showed significantly upregulated the mRNA level of KLF2 (data not shown). In conjunction with the western blot results presented in this study, which demonstrate increased KLF2 protein levels following simvastatin treatment, our findings suggest that simvastatin treatment may upregulate KLF2 expression at both the transcriptional and translational levels. In the present study, our findings demonstrate that simvastatin treatment significantly enriches the KLF2-positive population of breast cancer cells (Figure 9B). Future investigations could be focused on determining whether simvastatin treatment leads to apoptosis or cell cycle arrest specifically in KLF2-expressing cells, which would provide valuable direct evidence supporting KLF2 as a promising therapeutic target of simvastatin treatment.

In summary, our investigation explored the relationship between KLF2 expression and various pathological features, immune infiltration, angiogenesis, regulatory networks, and therapy in breast cancer using multiple perspectives. Our findings indicate that KLF2 acts as a tumor suppressor and is associated with diverse immune infiltration cells, potentially affecting the breast tumor immune microenvironment by enhancing dendritic cell activation. Thus, KLF2 may serve as a promising biomarker for breast cancer diagnosis and treatment. Nevertheless, our study relied heavily on bioinformatics analysis and partial *in vivo* and *in vitro* experiments. Therefore, further experimental validation is necessary to confirm these results and establish the role of KLF2 as a diagnostic marker and potential therapeutic target for breast cancer.

## Data Availability

The original contributions presented in the study are included in the article/Supplementary Material, further inquiries can be directed to the corresponding authors.
