# Supplementary figures and images for: KLF2 is a clinical diagnostic and treatment biomarker of breast cancer
Source: Front Cell Dev Biol. 2023 Apr 13;11:1182123. doi: 10.3389/fcell.2023.1182123 (PMC10133575; doi:10.3389/fcell.2023.1182123)

**Supplementary Figure1 The impact of KLF2 on the survival probability of pan-cancer patients.**


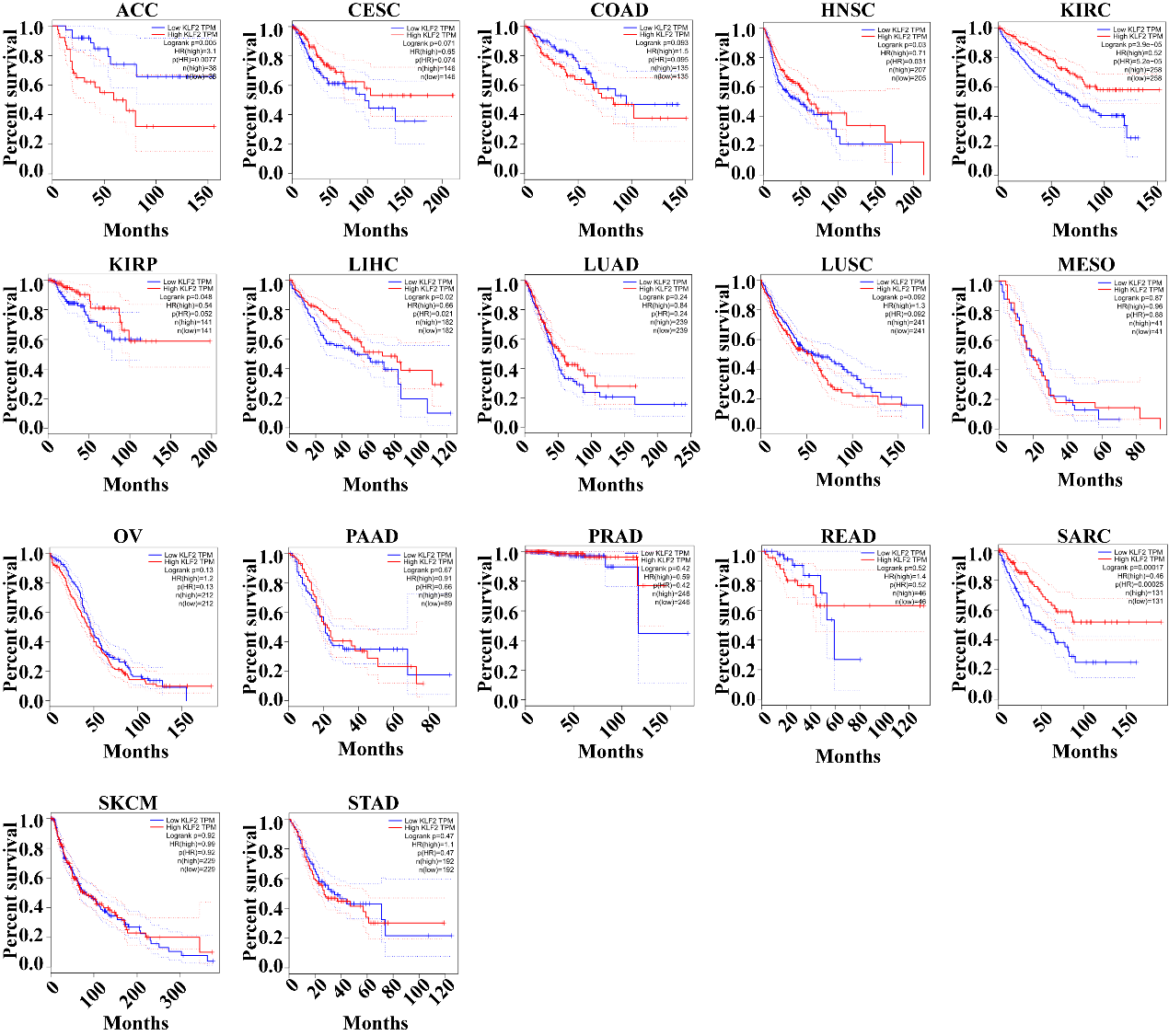

Supplement: Supplementary file 1 [file DataSheet1.docx]
